# Palmitoylethanolamide reduces pain-related behaviors and restores glutamatergic synapses homeostasis in the medial prefrontal cortex of neuropathic mice

**DOI:** 10.1186/s13041-015-0139-5

**Published:** 2015-08-12

**Authors:** F. Guida, L. Luongo, F. Marmo, R. Romano, M. Iannotta, F. Napolitano, C. Belardo, I Marabese, A. D’Aniello, D. De Gregorio, F. Rossi, F. Piscitelli, R. Lattanzi, A. de Bartolomeis, A. Usiello, V. Di Marzo, V. de Novellis, S Maione

**Affiliations:** Department of Experimental Medicine, Section of Pharmacology L. Donatelli, Second University of Naples, 80138 Naples, Italy; Department of Neuroscience, Laboratory of Molecular and Translational Psychiatry, University School of Medicine “Federico II”, Naples, Italy; Behavioural Neuroscience Laboratory, CEINGE - Biotecnologie Avanzate, Via Comunale Margherita 482, 80145 Naples, Italy; Institute of Biomolecular Chemistry, Consiglio Nazionale delle Ricerche, Via Campi Flegrei 34, 80078 Pozzuoli, NA Italy; Stazione Zoologica “Anton Dohrn”, Naples, Italy; Department of Women, Child and General and Specialistic Surgery, Second University of Naples, Naples, Italy; Department of Physiology and Pharmacology, Sapienza University of Rome, 00185 Rome, Italy

## Abstract

**Background:**

Enhanced supraspinal glutamate levels following nerve injury are associated with pathophysiological mechanisms responsible for neuropathic pain. Chronic pain can interfere with specific brain areas involved in glutamate-dependent neuropsychological processes, such as cognition, memory, and decision-making. The medial prefrontal cortex (mPFC) is thought to play a critical role in pain-related depression and anxiety, which are frequent co-morbidities of chronic pain. Using an animal model of spared nerve injury (SNI) of the sciatic nerve, we assess bio-molecular modifications in glutamatergic synapses in the mPFC that underlie neuropathic pain-induced plastic changes at 30 days post-surgery. Moreover, we examine the effects of palmitoylethanolamide (PEA) administration on pain-related behaviours, as well as the cortical biochemical and morphological changes that occur in SNI animals.

**Results:**

At 1 month, SNI was associated with mechanical and thermal hypersensitivity, as well as depression-like behaviour, cognitive impairments, and obsessive-compulsive activities. Moreover, we observed an overall glutamate synapse modification in the mPFC, characterized by changes in synaptic density proteins and amino acid levels. Finally, with regard to the resolution of pain and depressive-like syndrome in SNI mice, PEA restored the glutamatergic synapse proteins and changes in amino acid release.

**Conclusions:**

Given the potential role of the mPFC in pain mechanisms, our findings may provide novel insights into neuropathic pain forebrain processes and indicate PEA as a new pharmacological tool to treat neuropathic pain and the related negative affective states.

**Graphical Abstract:**
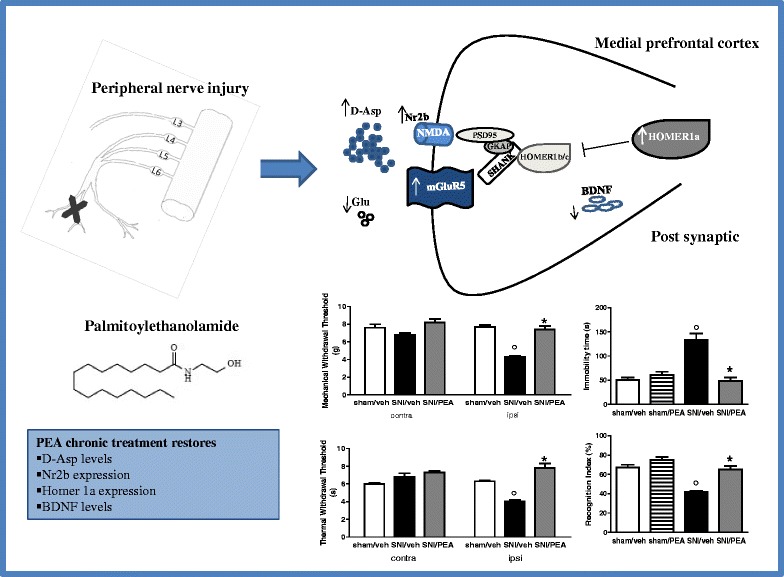
Palmitoylethanolamide: a new pharmacological tool to treat neuropathic pain and the related negative affective states.

**Electronic supplementary material:**

The online version of this article (doi:10.1186/s13041-015-0139-5) contains supplementary material, which is available to authorized users.

## Background

Accumulating evidence suggests that dysfunction of the glutamate system is correlated with the pathophysiological mechanisms responsible for chronic pain development or maintenance. Neuropathic or inflammatory injury triggers structural and functional changes in the peripheral or central sensory circuits, resulting in altered nociceptive signal processes, such as spontaneous pain, allodynia, and hyperalgesia [1–4]. Chronic pain can interfere with specific brain areas involved in glutamate-dependent neuropsychological features, such as cognition, memory, and decision-making [5, 6]. Enhanced glutamate release and over-activation of glutamate receptors following nerve injury has been observed in limbic and cortical areas involved in pain-related responses, including the anterior cingulate cortex (ACC), insular cortex, and prelimbic and infralimbic (PL-IL) cortices [7, 8]. Moreover, the activation of genes involved in glutamate-mediated cellular signalling, via *N*-methyl-D-aspartate (NMDA) or metabotropic glutamate receptors (mGluRs), has been described in cortical areas under chronic pain conditions [9–11]. In particular, the medial prefrontal cortex (mPFC) is thought to play a key role in pain-related depression and anxiety, which are frequent co-morbidities of chronic pain [12, 13]. In fact, neural reorganization and changes in neurotrophic or excitatory factors have been demonstrated in the mPFC during neuropathic pain [14, 7]. PL-IL cortex corresponds to the dorsal-lateral prefrontal cortex in primates. It plays a crucial role in pain processing and pathological pain deeply affects its synaptic organization [7, 8, 14]. NMDA-induced changes in dendritic plasticity has been shown in the PL-IL cortex of neuropathic rats [15], whereas cortex deactivation has been shown in rodents with persistent inflammatory pain [16].

The endocannabinoid (eCB) system has been involved in the pathogenesis of neuropathic pain [17–19]. In addition, functional cross-talk between the endocannabinoid/endovanilloid and glutamatergic systems in antinociception as well as in psychotic disorders has been identified [20–22]. Endogenous levels of eCB ligands change during neuropathic pain in several regions of ascending and descending pain pathways [23, 24]. In addition to the classical eCBs, endocannabinoid-related *N-*acylethanolamines, such as palmitoylethanolamide (PEA) and oleoylethanolamide (OEA), have been shown to participate in pain mechanisms [25, 19]. In particular, PEA, which is abundant in the central nervous system (CNS) in physiological conditions, has been shown to be involved in neuroprotective mechanisms activated under several pathological states, including neuropathic pain [26–28]. PEA is an endogenous anti-inflammatory mediator with pleiotropic effects due to the complex mechanisms of action that have not been clarified. Our previous studies, as well as those of others, have demonstrated that chronic administration of PEA reduces pain behaviours and counteracts spinal neuronal hyper excitability in murine models of persistent pain [29, 2].

This study aimed to investigate bio-molecular modifications in glutamate synapses of the mPFC that underlie neuropathic pain-induced plastic changes in an animal model of spared nerve injury (SNI) of the sciatic nerve at 30 days post-surgery. We evaluated (i) pain behaviours and the affective-cognitive impairments associated with persistent peripheral neuropathy, (ii) bio-molecular changes occurring in the proteins in mPFC synaptosomal fractions of SNI mice, (iii) changes in levels of amino acids and acylethanolamines (PEA and oleoylethanolamide, OEA) in the mPFC of SNI mice, and (iv) the effects of PEA administration on pain-related behaviours, as well as cortical biochemical and morphological changes in SNI animals.

## Methods

### Animals

Male CD-1 mice (30–35 gr) were housed 3 per cage under controlled conditions (12:12 h light/dark cycle; room temperature 20–22 °C, humidity 55–60 %) with chow and tap water were available ad libitum. The experimental procedures were permitted by the Animal Ethics Committee of the Second University of Naples. Animal care was in observance with the IASP and European Community (E.C. L358/1 18/ 12/86) guidelines on the use and protection of animals in experimental research. All efforts were made to reduce animal suffering and the number of animals used.

### Spared Nerve Injury (SNI)

Neuropathy condition was induced according to Decostered and Woolf method [30]. Mice were anesthetized [ketamine (60 mg/kg) + xylazine (10 mg/kg)] and the sciatic nerve was exposed. The tibial and common peroneal nerves were ligated and then transected, leaving the sural nerve intact. Sham animals were anaesthetized and the sciatic nerve was exposed, but not transected.

### Pain behaviour

#### Plantar test

Thermal hyperalgesia was evaluated using the plantar test (Ugo Basile). Mice were placed in clear Plexiglas chambers; a mobile infrared radiant heat source was focused under the plantar surface of the hind paws. Latency to paw withdrawal was determined by a timer. The stimulus intensity was preset to obtain a paw withdrawal latency ranging from 9 to 11 s. Withdrawal threshold of the hind paws was defined as the mean of three measurements. *Dynamic Aesthesiometer.* Mechanical allodynia was evaluated using the Dynamic Plantar Aesthesiometer (Ugo Basile). This equipment employs a single non-flexible filament (0.5-mm diameter) to apply an increasing force to the plantar surface of the mouse hind paw. Animals were placed in a cage with a wire mesh floor and allowed to acclimatize before testing. The filament was applied to the plantar area (plantar territory of the sural nerve) of the hind paw and it began to exert an increasing upward force, reaching a maximum of 30 g in 10 s, until the paw was withdrawn. The withdrawal threshold was defined as the force, in grams, at which the mouse withdrew its paw. Withdrawal thresholds of ipsilateral and contralateral paws were determined three times, and the reported value is the mean of the three evaluations. Pain behaviors were evaluated following PEA or OEA (10 mg/kg, i.p.) repeated treatment (15 days) in sham or SNI mice (up to 30 days). In a separate set of experiments, sham and SNI mice were used for the assessment of mechanical allodynia 30 days after surgery before and after a single intra-PL-IL cortex microinjection of 200 nL of vehicle (0.5 % DMSO in artificial cerebrospinal fluid, ACSF, in mM: 125.0 NaCl, 2.6 KCl, 2.5 NaH2PO4, 1.3 CaCl2, 0.9 MgCl2, 21.0 NaHCO3, and 3.5 glucose, oxygenated and equilibrated to pH 7.4), or PEA or OEA (6 nmol/mouse). The surgical preparation for intra-PL-IL cortex administration was performed the day before the experiment through a 20-gauge stainless steel guide cannula stereotaxically lowered, by applying coordinates from the atlas of Paxinos and Watson (1986) (AP: 1.4 mm, L: 0.5 mm from bregma, V: 3 mm below the dura) [31]. The drugs doses were chosen based on our previous studies [2, 25].

#### Tail suspension

Animals were suspended by the tail on a horizontal bar (50 cm from the floor) using adhesive tape placed 4 cm from the tip of the tail. The duration of immobility was recorded in seconds over a period of 6 min of habituation. Immobility time was defined as the absence of escape-directed behaviour.

#### Object recognition

The experiment started with the habituation period, during which mice were allowed to freely explore for 1 h the apparatus which consists of a rectangular open box (40 × 30 × 30 cm width × length × height) made of grey polyvinyl chloride (PVC) illuminated by a dim light. The day after each mouse was allowed to explore two identical objects positioned in the back left and right corners for 5 min (acquisition). A camera recorded the time spent on exploration of each object. In the test trial, which was carried out for 2 h after the acquisition, one of the two objects was replaced with a new different object. The time spent exploring the object was the time the mouse spent with its nose directed, and within 1 cm, from the object. The behaviour of mice was analyzed by an observer blind to the treatment. Data were expressed as percentage of recognition index (RI %), which was calculated as the percentage of the time spent exploring the novel object / time spent exploring the novel object + time spent exploring the familiar object × 100.

#### Marble-burying

Mice were individually placed in a cage (21 × 38 × 14 cm length x width x height) containing 5 cm layer of sawdust bedding and fifteen glass marbles (1.5 cm in diameter) arranged in three rows. Mice were left undisturbed for 15 min under dim light. An observer blind to the treatment counted the time spent in digging behaviour and the number of marbles buried (at least two or third buried in the sawdust). At the end of the test the animal was removed to its own cage.

### Western blotting synaptosome

Synaptic protein expression in prefrontal cortex was assessed by western blotting analysis. The dissected prefrontal cortex tissue samples were lysed in Syn-PER Synaptic Protein Extraction Reagent (Thermoscientific, USA) (10 ml of Syn-PER Reagent per gram of tissue), in order to isolate synaptosomes. Lysis buffer contained a cocktail of phosphatase and proteinase inhibitors, Halt Protease Inhibitor Cocktail (Thermoscientific, USA), added immediately before use to avoid proteins degradation. Homogenization was carried out by mean dounce tissue grinder on ice and homogenate was centrifuged at 1200×*g* for 10 min at 4 °C. The pellet was removed and supernatant was centrifuged at 15000×*g* for 20 min at 4 °C. After removing supernatant containing cytosolic fraction, synaptosome pellet was suspended adding 1–2 ml of Syn-PER Reagent per gram of sample. Protein concentration in each sample was determined using Bradford assay and equal amounts of total proteins were loaded onto 10 % polyacrylamide gel for Psd-95, Shank1, mGluR5, mGluR7, NR2a and b, Trkb, 15 % polyacrylamide gel for Homer1a and Homer1b. Proteins were separated by SDS-PAGE and transferred to nitrocellulose blotting membranes (GE Healthcare, UK). Following blocking in buffer solution including 5 % milk and 0.1 % Tween-20 in TBS (Tris HCl 25 mM, NaCl 137 mM), the membranes were immunoblotted with the following primary antibodies: Homer1a (1:200; Santa Cruz Biotechnology), Homer1b (1:1000, Santa Cruz Biotechnology), Psd95 (1:1000, Santa Cruz Biotechnology), Shank1 (1:1000, Santa Cruz Biotechnology), mGluR5 (1:1000, Santa Cruz Biotechnology), NR2a (1:1000, Santa Cruz Biotechnology), NR2b (1:1000, Santa Cruz Biotechnology), Trkb (1:1000, Santa Cruz Biotechnology). Then blots were incubated in species-appropriate horseradish peroxidase-conjugated secondary antibodies (1:2000, Santa Cruz Biotechnology) and proteins of interest visualized by ECL detection (LiteAblot extend, Euroclone) at molecular weight target. Quantification of the bands intensity on scanned filters was achieved by Quantity one analysis software (Bio-Rad, USA). Background value was subtracted to minimize variability across membranes and each lane was normalized for the corresponding actin value for variation in loading and transfer. Data were analyzed by one-way ANOVA followed by Turkey’s post-hoc test (level of significance *p* < 0.05). Data were expressed as percentage of the mean of controls. Statistical analyses were performed with StatView software (version 5.0.1.0; SAS Institute).

### Western blotting tissue

After protein determination by Bio-Rad Protein Assay kit (Bio-Rad), equal amounts of total proteins (30 μg) for each prefrontal cortex sample were loaded onto 10 % polyacrylamide gels. Proteins were separated by SDS-PAGE and transferred overnight to membranes (Immobilon PVDF Membrane, Millipore). The membranes were immunoblotted overnight using selective antibodies against BDNF (diluted 1:500, Santa Cruz Biotechnology, Dallas, Texas), pSer473-Akt1, pSer235/236-S6, pSer-240/244-S6 (each diluted 1:500, Cell Signaling Technology, Beverly, MA). Blots were then incubated with appropriate horseradish peroxidase-conjugated secondary antibody and target proteins were visualized by ECL detection (GE Healthcare), followed by quantification through Quantity One software (Bio-Rad). Antibodies against Akt1 and S6 (both diluted 1:500, Cell Signaling Technology, Beverly, MA), that are not phosphorylation state-specific were used to estimate the total amount of proteins. All optical density values were normalized using an antibody against GAPDH (1:5000, Santa Cruz Biotechnology, Dallas, Texas). Blots were then incubated in horseradish peroxidase-conjugated secondary antibodies and target proteins visualized by ECL detection (Pierce, Rockford, IL), followed by quantification by Quantity One software (Biorad). Normalized values were averaged and used for statistical comparisons performed by one-way ANOVA, followed by Turkey’s post-hoc test .

#### Cell cultures

Cortices of 17 days embryonic (E-17) mice were prelieved, dissected carefully in ice, typsinized in 0.25 % Trypsin-EDTA for 20 min. Tissues were centerfuged (2000 rpm) for 1 min and filtered. Cells were seeded in Poly D-Lysine- overnight pre-treated multiwells in serum free medium (Neurobasal Medium + 2 % B27 growth factor). Cells were left to grow by changing half of medium every 3 days and then they were treated at 14 DIV.

#### Cultured neurons western blotting

Total lysates from neuron cultures obtained through RIPA buffer lysis were analyzed by western blot experiment after treatment with PEA (100 nM, 24 h). Denaturated protein (50 μg) was loaded on 10 % polyacrilamide gel and transferred on a Polyvinylidene Fluoride (PVDF) membrane incubated overnight at 4 °C with the horseradish peroxidase conjugated antibodies: goat polyclonal anti-mGluR5 (1:500; Santa Cruz Biothecnology), rabbit polyclonal anti-Nr2b (1:500; Abcam), goat polyclonal anti-NR2a (1:1000; Santa Cruz Biothecnology), and then with the relative secondary antibody for 1 h. Reactive bands were visualized on a X-ray film. Whereas the same membrane trip was used for revealing the expression of more than one protein of interest, a mild stripping at 60 °C for 10 min was done. Monoclonal anti-β-tubulin antibody (1:1000; Sigma) was used as housekeeping protein to check for identical protein loading. Images were captured, stored, and analyzed with the Quantity One software (BioRad, Hercules, CA).

#### *In vivo* single unit extracellular recordings

Mice for electrophysiological recordings were anaesthetized with pentobarbital (50 mg/kg, i.p.) and placed in a stereotaxic device (David Kopf Instruments, Tujunga, CA). Body temperature was maintained at 37 ° C with a temperature-controlled heating pad. In all surgical preparations, the scalp was incised and holes were drilled in the skull overlying the site of recording, medial prefrontal cortex (AP: +1–2.9, L: 0.2–0.3 from bregma and V: 1.2–3 mm below dura) and contralateral (right) with respect to the nerve insult (left). Anaesthesia was maintained with a constant continuous infusion of propofol (5–10 mg/kg/h, i.v.). A glass-insulated tungsten filament electrode (3–5 MΏ) (FHC Frederick Haer & Co., ME) was stereotaxically lowered into the mPFC and the recorded signals were amplified and displayed on a digital storage oscilloscope to ensure that the unit under study was unambiguously discriminated throughout the experiment. Signals were processed by an interface CED 1401 (Cambridge Electronic Design Ltd., UK) and analyzed through Spike2 software (CED, version 4) to create peristimulus rate histograms online and to store and analyze digital records of single-unit activity off-line. Configuration, shape, and height of the recorded action potentials were monitored and recorded continuously. Neurons showed a cluster of spikes with an increased frequency showing typically a Gaussian pattern of distribution after stimulation. We measured the duration of the excitation (in seconds) as the period of the increased firing activity which exceeds the average baseline value +2 standard deviations (SDs). Moreover, we measured the the onset of excitation (in milliseconds) which was considered as the time from the application of the stimulus (artifact) to the first-evoked spike which exceeds the average baseline value +2 SD. Moreover, the EAP slope, indicating rate of activation of synaptic receptors, was used for the evaluation of the efficacy of synaptic transmission. Electrophysiolgical evaluations were performed in the following groups of mice (*n* = 10): 1) sham mice; 2) 15 or 30 days SNI mice.

### Microdialysis

Microdialysis experiments were performed in awake and freely moving mice. In brief, mice were anaesthetized and stereotaxically implanted with concentric microdialysis probes into the mPFC using coordinates: AP: 1.4–1.8 mm, L: 0.3–05 mm from bregma and V: 3.0 mm below the dura. Microdialysis probes were constructed with 22G (0.41 mm I.D., 0.7 mm O.D.) stainless steel tubing: inlet and outlet cannulae (0.04 mm I.D., 0.14 mm O.D.) consisted of fused silica tubing. The probe had a tubular dialysis membrane (Enka AG, Wuppertal, Germany) 1.3 mm in length. Following a recovery period of 24 h, dialysis was commenced with ACSF (NaCl 147 mM, CaCl_2_ 2.2, KCl 4 mM; pH 7.2) perfused at a rate of 1 μL/min by a Harvard Apparatus infusion pump. Following a 60-min equilibration period, 12 consecutive 30-min dialysate samples were collected. At the end of experiments, mice were anaesthetized and their brains perfused fixed via the left cardiac ventricle with heparinised paraformaldehyde saline (4 %). Brains were dissected out and fixed in a 10 % formaldehyde solution for 2 days. The brain were cut in 40-μm thick slices and observed under a light microscope to identify the probe locations. Dialysates were analyzed through an high performance liquid chromatography method. The system comprised a Varian ternary pump (mod. 9010), a C18 reverse-phase column, a Varian refrigerated autoinjector (mod. 9100), a Varian fluorimetric detector. Dialysates were precolumn derivatized with Opthaldialdehyde (10 μl dialysate + 10 μl o-pthaldialdehyde) and amino acid conjugates resolved using a gradient separation. The mobile phase consisted of 2 components: 1) 0.2 M sodium phosphates buffer (pH 5.8), 0.1 M citric acid (pH 5.8) and 2) 90 % acetonitrile, 10 % distilled water. Additionally, to determine the amount of area peak due to D-Aspartate, a parallel sample was incubated with 2 ml of purified D-Asp Oxidase for 15 min at 37 °C and chromatographed as above. The total disappearance or the reduction of area peak corresponding to D-Asp elution peak confirmed the presence of D-Asp and gave the exact amount of the content of D-Asp. A typical analysis is represented in Fig. 5. Data were collected by a Dell Corporation PC system 310 interfaced by Varian Star 6.2 control data and acquisition software. The data were expressed as mean of five samples for each mice.

#### Measurement of N-acylethanolamines levels (PEA and OEA)

The extraction, purification and quantification of N-acylethanolamines from tissues has been performed as previously described [32]. Briefly, tissues were dounce-homogenized and extracted with chloroform/methanol/Tris–HCl 50 mmol/l pH 7.5 (2:1:1, vol/vol) containing internal standards [2H]4 PEA and [2H]2 OEA 50 pmol each). The lipid-containing organic phase was dried down, weighed, and pre-purified by open-bed chromatography on silica gel. Fractions were obtained by eluting the column with 99:1, 90:10 and 50:50 (v/v) chloroform/methanol. The 90:10 fraction was used for PEA and OEA quantification by liquid chromatography—atmospheric pressure chemical ionization—mass spectrometry by using a Shimadzu high-performance liquid chromatography apparatus (LC-10ADVP) coupled to a Shimadzu (LCMS-2020) quadrupole mass spectrometry via a Shimadzu atmospheric pressure chemical ionization interface as previously described [32[. LC analysis was performed in the isocratic mode using a Discovery C18 column (15 cm × 4.6 mm, 5 μm) and methanol/water/acetic acid (85:15:1 by vol.) as mobile phase with a flow rate of 1 ml/min. The amounts of componds in tissues, quantified by isotope dilution with the abovementioned deuterated standards, are expressed as pmol/mg.

#### Immunohistochemistry and immunofluorescence

Under pentobarbital anesthesia (50 mg/kg, i.p.), animals were transcardially perfused with saline solution followed by 4 % paraformaldehyde in 0.1 M phosphate buffer. The brains were excised, post fixed for 3 h in the perfusion fixative, cryoprotected for 72 h in 30 % sucrose in 0.1 M phosphate buffer, and frozen in Optimal cutting temperature (O.C.T.) embedding compound. Transverse sections (20 μm) were cut using a cryostat and thaw-mounted onto glass slides. Slides were incubated overnight with primary antibody solutions for the microglial cell marker Iba-1 (rabbit anti-ionized calcium binding adapter molecule-1; 1:1000; Wako Chemicals, Germany). Possible non-specific labeling of mouse secondary antibody was detected by using secondary antibody alone. Following incubation, sections were washed and incubated for 2 h with secondary antibody solution (donkey anti-rabbit Alexa FluorTM 488; 1:1000; Molecular Probes, USA). Slides were washed, coverslipped with Vectashield mounting medium (Vector Laboratories, USA), and visualized under a Leica fluorescence microscope. The number of cells positive for Iba-1 was determined within a box measuring 2×10^4^ μm^2^ that was placed in the m-PFC. Six sections were assessed from one animal and three animals were used for each group. To avoid cell over-counting, only DAPI-counterstained cells were considered as positive profiles. Iba-1 -positive cells were identified as resting (with small somata bearing long, thin and ramified processes), activated microglia (with hypertrophy together with retraction of processes to a length shorter than the diameter of the somata) or hyper-branched microglia (with increased number of branches).

## Results

### Effect of systemic treatment with PEA (or OEA) on pain behaviour

**Fig. 1:**
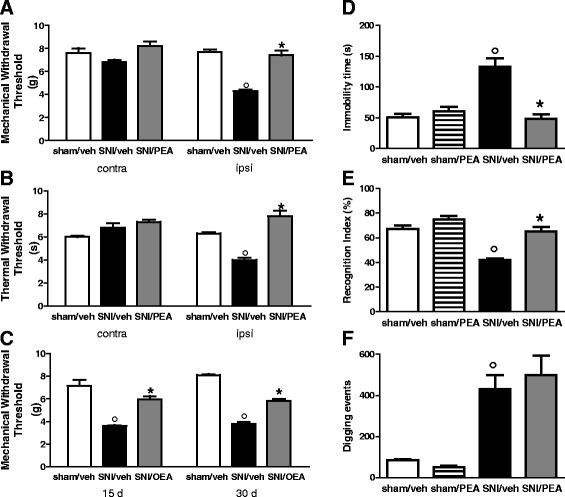
Effects of PEA (or OEA) on mechanical allodynia (**a** and **c**), on thermal hyperalgesia (**b**), on duration of immobility in the tail suspension (**d**), on the index of recognition in the novel object recognition task (**e**) and on on the number of digging events in the marble burying (**f**) in sham and SNI mice. Behaviours were determined 30 days after SNI or sham surgery, in presence of vehicle (Pluronic F-68, 5 % v/v) or PEA or OEA (10 mg/kg). Each point represents the mean ± standard error of the mean (S.E.M) of 10–12 mice. ° and * indicate significant differences compared to sham/vehicle and SNI/vehicle, respectively. *P* < 0.05 was considered statistically significant

Spared nerve injury (SNI) of the sciatic nerve decreased the ipsilateral paw nociceptive thresholds to mechanical (4 g ± 0.1) or thermal stimuli (4.3 s ± 0.2) (Fig. 1a and b). No change was observed in mechanical or thermal thresholds in the contralateral paw of SNI or sham-operated mice. We found that repeated PEA treatment (10 mg/kg i.p., once per day) significantly increased both the thermal and mechanical thresholds in SNI mice (7.8 s ± 0.5 and 7.4 g ± 0.4, respectively) 30 days after injury. Moreover, no changes in pain thresholds of PEA-treated sham animals were observed (not shown). Similarly to PEA, repeated OEA treatment (10 mg/kg i.p., once per day) significantly increased mechanical threshold in SNI mice (5.96 ± 0.2 and 5.82 ± 0.19, respectively) at 15 and 30 days after the nerve injury (Fig. 1c). Whilist, no change in pain thresholds of OEA-treated sham animals was observed (not shown).

### Effect of systemic treatment with PEA on tail suspension immobility

Neuropathic mice show an increased duration of immobility during tail suspension 30 days after surgery. Indeed, the time of immobility was significantly higher in the SNI mice that received vehicle as compared to vehicle-treated sham animals (133.2 s ± 13.2 and 54.4 s ± 5.6, respectively). However, the repeated PEA treatment (10 mg/kg i.p., once per day) significantly reduced the time of immobility (48.5 s ± 7.4). PEA treatment did not change the time of immobility in sham mice (Fig. 1d).

### Effect of systemic treatment with PEA on the object recognition test

In the object recognition test SNI mice that received chronic vehicle administration showed a significant decrease in the recognition index (41.9 ± 1.2) as compared to the sham animals (67.10 ± 2.90) (Fig. 1e). The repeated PEA treatment (10 mg/kg i.p., once per day) significantly increased the recognition index in the SNI mice (65.0 ± 3.7), but not in the sham mice (74.9 ± 3.1).

### Effect of systemic treatment with PEA on marble-burying test

During the 15 min marble burying task, vehicle-treated sham and SNI mice significantly differed in the number of buried marbles 30 days after surgery, which was 6.6 ± 0.53 and 11.8 ± 0.37 respectively in sham and SNI mice. In addition, the number of digging events was significantly higher in SNI mice (431.4 ± 67.7) as compared to sham mice (84.40 ± 4.7) (Fig. 1e). We observed that the number of buried marbles (10.2 ± 0.41) and the number of digging events was not changed following PEA treatment, in both sham and SNI mice (Fig. 1f).

### Effect of SNI on acylethanolamines (PEA and OEA) in the mPFC

We next measured PEA and OEA level in the m-PFC of sham or SNI mice. We found that SNI increased PEA level (2.2 ± 0.3 pmol/mg) 15 days after the nerve injury, as compared with sham animals (0.9 ± 0.1 pmol/mg) (*P* < 0.05). However, endogenous PEA amount resulted normalized 30 days post SNI (1.1 ± 0.1 pmol/mg). The same surgical procedure did not affect OEA levels (0.8 ± 0.1 pmol/mg) in the mPFC.

### Effect of PEA on post-synaptic proteins expression in m-PFC synaptosomes in SNI mice

**Fig. 2:**
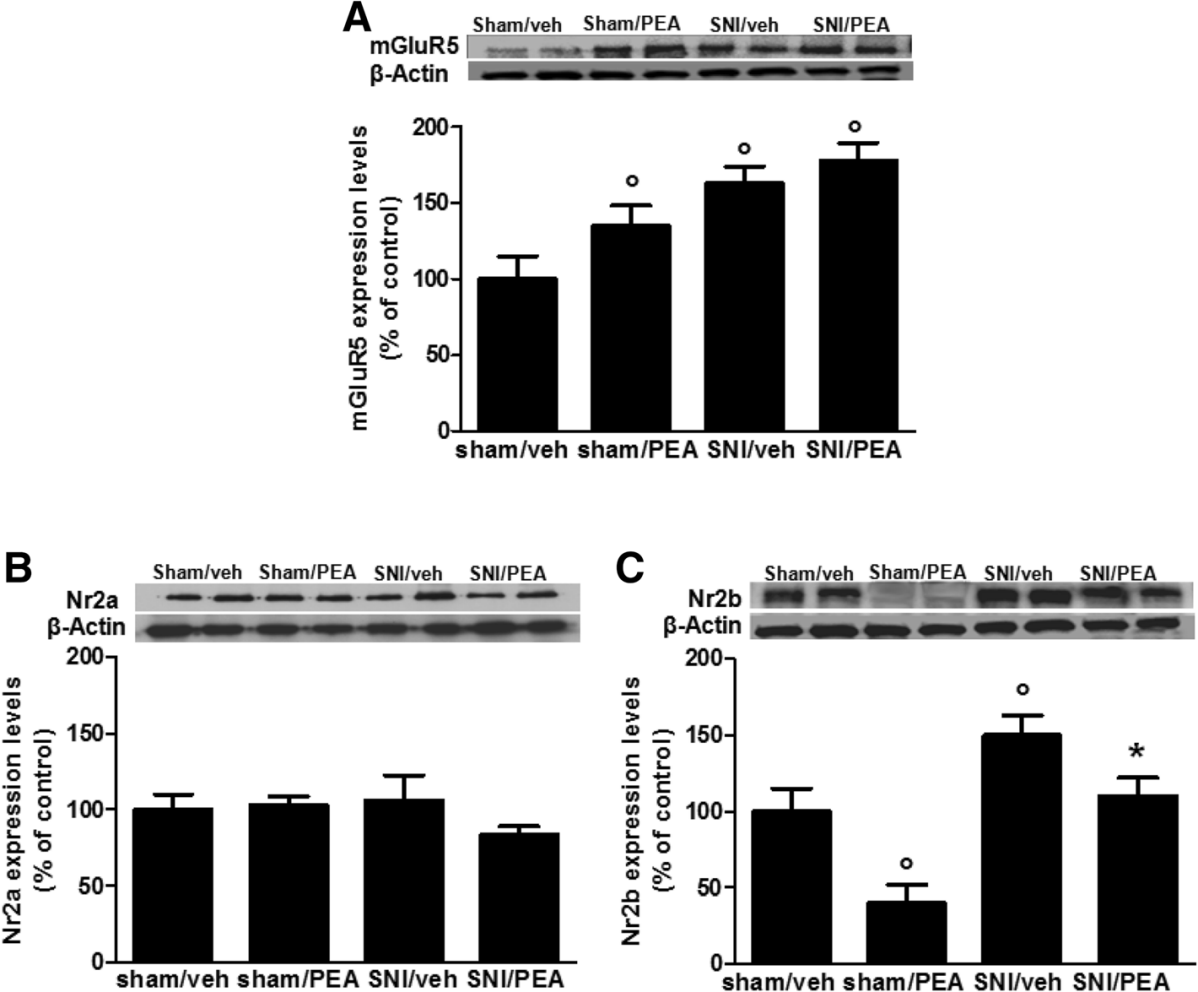
Expression levels of postsynaptic proteins, measured using Western blot analysis, in synaptosomes from the mPFC of sham and SNI 30 days after surgery, in presence of vehicle or PEA (10 mg/kg). “**a**”, “**b**” and “**c**” show the protein expressions of mGluR5, Nr2a and Nr2b, respectively, normalized to β-Actin. Data are represented as a mean ± SEM of 6 mice per group. ° and * indicate significant differences compared to sham/vehicle and SNI/vehicle, respectively *P* < 0.05 was considered statistically significant. ANOVA, post hoc Tukey

**Fig. 3:**
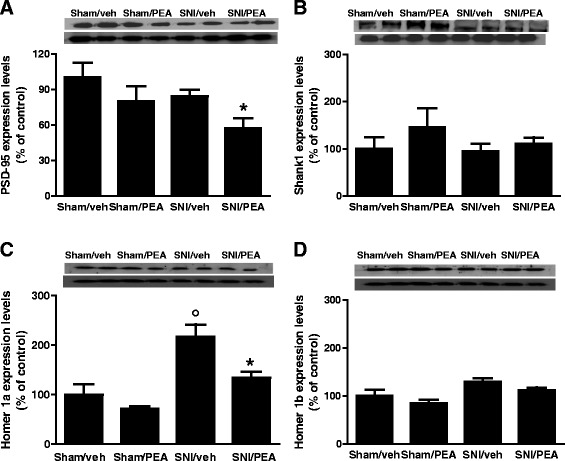
Expression levels of postsynaptic proteins, measured using Western blot analysis, in synaptosomes from the mPFC of sham and SNI 30 days after surgery, in presence of vehicle or PEA (10 mg/kg). “**a**”, “**b**”, “**c**” and “**d**” show the protein expression of PSD-95, SHANK-1, Homer-a and Homer-1b, respectively, normalized to β-Actin . Data are represented as a mean ± SEM of 6 mice per group. ° and * indicate significant differences compared to sham/vehicle and SNI/vehicle, respectively *P* < 0.05 was considered statistically significant. ANOVA, post hoc Tukey

Western blotting were carried on m-PFC synaptosomes fractions obtained from sham or neuropathic mice in the presence of vehicle or PEA (10 mg/kg, once per day). We found that 30 days of SNI induced significant changes in mGluR5, NMDAR-NR2b subunit and in the inducible homolog Homer 1a (Homer 1a) protein expression levels (Fig. 2). In particular, the mGluR5 protein levels resulted significantly increased in SNI mice, as well as, in PEA-treated SNI or sham animals (Fig. 2a). The SNI procedure also led to a significant increase in NR2b protein expression as compared with sham mice (Fig. 2c). This effect was completely reversed in SNI mice receiving PEA repeated treatment, and, interestingly, PEA also reduced NR2b expression in physiological condition (sham mice) (Fig. 2c). No changes in the NR2a subunit protein levels were observed (Fig. 2b). Moreover, SNI mice showed increased the Homer1a protein levels, which resulted significantly reduced in the PEA-treated animals (Fig. 3c). In contrast, the levels of Homer1b, which is constitutively expressed in the brain, did not change in neuropathy condition (Fig. 3b). Finally, PEA caused a decrease of post-synaptic density protein-95 (PSD-95) levels, which instead resulted unchanged in SNI mice (Fig. 3a). Any change in Shank-1, the protein bridge between NMDAR and other scaffolding proteins, in sham or SNI mice was observed (Fig. 3b).

### Effect of PEA on BDNF/TrkB/Akt signalling in m-PFC of SNI mice

**Fig. 4:**
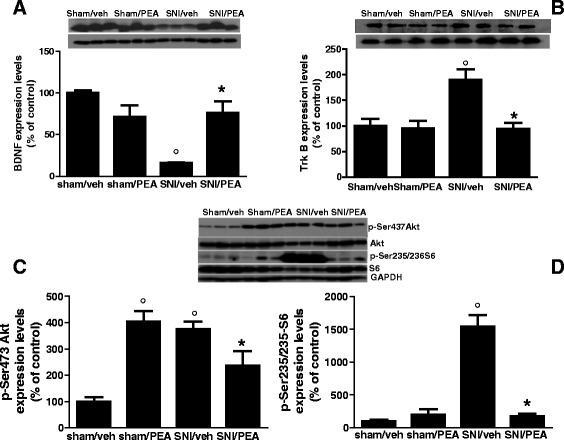
Expression protein levels measured using Western blot analysis, the mPFC of sham and SNI 30 days after surgery, in presence of vehicle or PEA (10 mg/kg). “**a**”, “**b**”, “**c**” and “**d**” show the protein expression of BDNF, Trk-B, p-Akt and p-S6 respectively, normalized to GAPDH. Data are represented as a mean ± SEM of 6 mice per group. ° and * indicate significant differences compared to sham/vehicle and SNI/vehicle, respectively. *P* < 0.05 was considered statistically significant. ANOVA, post hoc Tukey

To investigate possible changes in the cortical brain-derived neurotrophic factor (BDNF) regulation, we analysed the BDNF and its conjugate tropomyosin receptor kinase B (TrkB) level expression in the mPFC of SNI mice. Our data revealed a decrease in BDNF levels of SNI mice, as compared with sham animals. PEA treatment (10 mg/kg, once per day) significantly prevented this effect (Fig. 4a). Conversely, SNI mice showed increased TrkB expression which was significantly restored by PEA treatment (Fig. 4b). Finally, PEA was effective in restoring the protein level of the 70-kDa ribosomal S6 kinase (pS6K) and the serine/threonine kinase (Akt) which resulted increased in SNI mice. Interestingly, PEA also increased Akt expression in physiological condition (sham mice) (Fig. 4c and d).

### Effect of single microinjection of PEA (or OEA) in mPFC of SNI mice

**Fig. 5:**
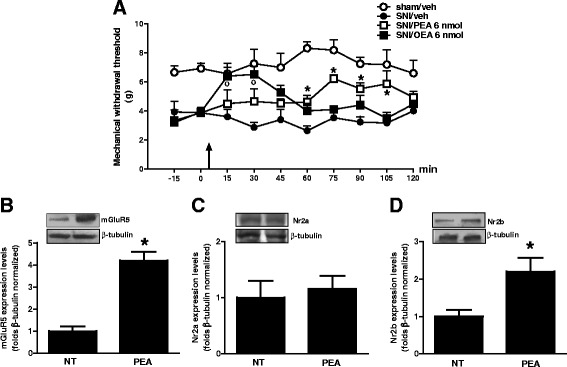
Effects of m-PFC microinjection of PEA or OEA (6 nmol/mouse) or vehicle (DMSO/ACSF, 0.05 %, v/v) on mechanical allodynia (**a**) in SNI mice 30 days after surgery. Each point represents the mean ± standard error of the mean (S.E.M) of 6–7 mice. Black arrows indicates drug injection. * and ° indicate significant differences of PEA or OEA treatment, respectively compared to SNI/vehicle. *P* < 0.05 was considered statistically significant. “**b**”, “**c**” and “**d**” show the protein expression of mGluR5, Nr2a and Nr2b, respectively, normalized to β-Tubulin. Data are represented as a mean ± SEM of 4–5 samples per group. * indicates significant differences compared to no treated cells. *P* < 0.05 was considered statistically significant. ANOVA, post hoc Tukey

Single microinjection of PEA or OEA (6 nmol/mouse) into the mPFC led to a reduction of mechanical allodynia in SNI mice 30 days post injury. In particular, we observed that single PEA microinjection decreased mechanical threshold with maximum effect at 75 min post-drug (6.2 g ± 0.26) with respect to SNI/vehicle mice (3.5 g ± 0.4) (Fig. 5a). On the other hand, OEA treatment immediately and transiently reduced mechanical allodynia (6.4 g ± 0.54) which lasted up to 30 min post injection (Fig. 5a). At the dose tested, PEA or OEA did not change the pain thresholds in both sham or SNI animals (not shown).

### Effect of PEA on post-synaptic proteins expression in cultured cortical neurons

To assess a possible specific effect on neuronal cells, we applied PEA in cultured neurons obtained from cortices of pups mice. We observed that PEA exposure (100 nM, 24) was capable to modify part of the post-synaptic proteins expression that we found changed in PEA-treated animals. In particular, we found an increase in the expression level of mGluR5 and in the inducible NMDA-NR2b subunit as compared with not treated cells, while no changes in the NR2a levels were observed. (Fig. 5b, c and d).

### Electrophysiological characterization of mechanical stimulation-evoked responses of mPFC (+) neurons in sham, SNI 15 days and SNI 30 days mice

**Fig. 6:**
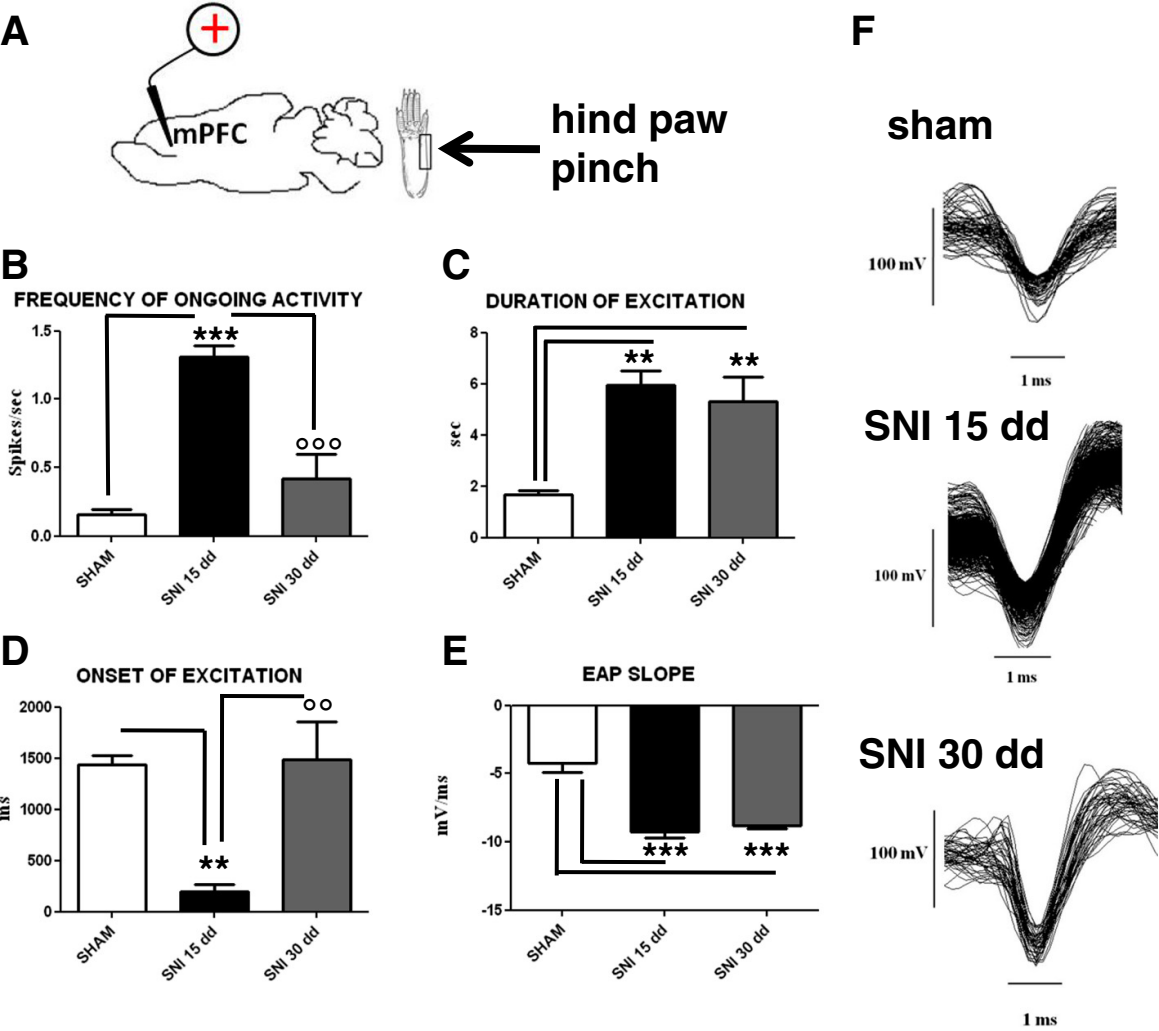
The figure shows different parameters of mechanical stimulus-evoked excitation in sham and SNI mice, 15 and 30 days (dd) after the induction of SNI. “**a**” shows schematic illustration of mechanical stimulation applied to the hind paw (contralateral to the mPFC). “**b**”, “**c**” and “**d**” show the mean ± standard error of the spontaneous activity, the duration and the onset of excitation in the different groups of mice (*n* = 10). “**e**” shows relative EAP slopes.* indicates statistically significant difference versus sham mice. ° indicates significant difference versus SNI 15 days mice. *P* < 0.05 has been considered as value of significance. The double symbol indicates *P* < 0.01 and the triple symbol indicates *P* < 0.001. “**f**” shows representative spike-waveform of mPFC (+) neurons recorded in sham, SNI 15 days and SNI 30 days mice

Single-unit extracellular recording in anesthetized mouse was made from individual neurons in the mPFC. The slow spontaneous firing rate of about 0.1–1.5 ± 0.4 spikes/s from recorded neurons was consistent with presumed pyramidal cells [33, 34]. (Fig. 6a) rather than fast-spiking interneurons, the latter having a higher baseline firing rate (>10 Hz) and narrower spike waveform (<300 μs) [35, 36]. We investigated the mPFC neurons with ongoing activity that responded with excitation [mPFC(+)] to mechanical stimulation by von Frey filaments with bending force of 97.8 mN (noxious stimulation) for 2 s [37]. Neurons that displayed no change in firing in response to stimulation were discarded. The duration and the onset of excitation were 1.67 ± 0.16 s and 1433 ± 94 ms, respectively (Fig. 6c and d), while the value of slope was −4.22 ± 0.67 mV/ms (Fig. 6e). 14 days-SNI mice showed an increased ongoing activity [1.31 ± 0.08 spikes/s; *P* < 0.001], as compared to sham animals. Moreover, we also observed an increase in the duration of the excitation [5.94 ± 0.57 s; *P* < 0.01] (Fig. 6d) and in the EAP slopes [−9.22 ± 0.48 mV/ms] (Fig. 6e), while the onset was reduced [193.6 ± 77.18 ms; *P* < 0.01] (Fig. 6e). Intriguingly, the ongoing cell activity, as well as, the onset of excitation resulted normalized in 30 days SNI mice [0.42 ± 0.17 spikes/s; *P* < 0.01 and 1486 ± 370.2 ms; *P* < 0.05, respectively] (Fig. 6c and e). However, the duration of the excitation [5.32 ± 0.93 s, *P* < 0.01 versus sham] and the EAP slopes [−8.77 ± 0.22 mV/ms, respectively; *P* < 0.001 versus sham] did not show any difference as compared with 15 days-SNI mice.

### PEA effects on amino acids release in the mPFC of SNI mice

**Fig. 7:**
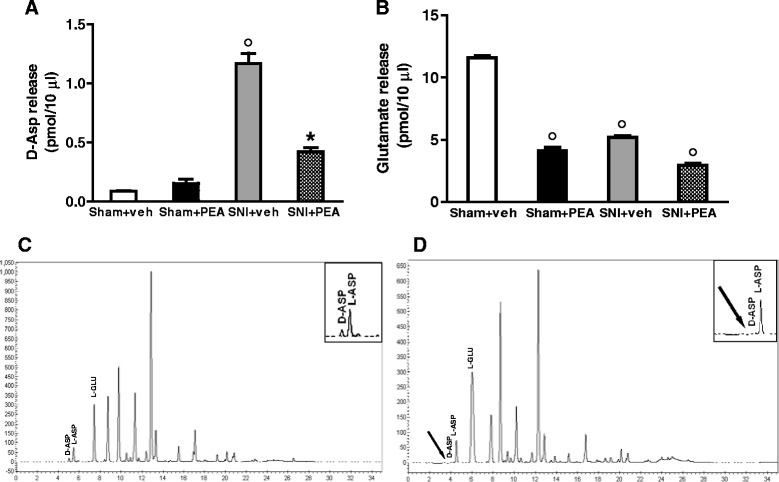
Release of D-Aspartate (**a**) or glutamate (**b**) in sham or SNI mice 30 days after injury. The values of extracellular amino acids in the mPFC were expressed as pmol in 10 μl of perfusate. Each point represents the mean ± S.E.M of 7–8 animals per group. *P* < 0.05 was considered statistically significant. ANOVA, post hoc Tukey. “**c**” shows a typical HPLC determination of D-Asp, L-Asp and Glutamate in neuropathic mice. “**d**” shows the same sample as in A after treatment with DDO. The peak corresponding to D-Asp disappears because of oxidation by DDO. The arrow shows the elution of D-Asp

*In vivo* microdialysis methodology was used to assess the amino acids contents in the m-PFC of 30 days SNI mice. HPLC analysis revealed an important increase of extracellular D-Aspartate (D-Asp) levels in the mPFC of neuropathic mice as compared with sham animals (Fig. 7a). Accordingly, the D-Asp precursor, L-Aspartate (L-Asp), was also increased (not shown). On the contrary, glutamate levels resulted significantly decreased in SNI mice, but PEA was not able to revert this effect. Interestingly, we found that PEA also reduced glutamate levels in the mPFC of sham mice (Fig. 7b). The same surgical procedure did not affect dialysate GABA level (not shown).

### Effect of systemic PEA treatment on m-PFC microglia activation

To investigate the potential involvement of supraspinal microglia in mediated-PEA effects, we evaluated possible changes in cell morphology in the m-PFC of sham or SNI mice in presence of vehicle or PEA (10 mg/kg, i.p.). SNI did not change the number of both total or activated Iba-1 positive cells in the m-PFC of neuropathic rodents accordingly with previous findings [38]. However, PEA-treated sham animals showed an increase of the number of hyper-branched microglia with very long ramified processes as compared with vehicle-treated animals (Additional file 1).

## Discussion

Altered glutamate signalling strongly correlates with the pathophysiological processes responsible for chronic pain and development of neurological disorders. In addition to pain sensory symptoms, glutamate-driven neuro-psychological functioning is thought to be affected by chronic pain, and a complex forebrain network (the “pain matrix”) may represent the substrate for negative affective states (fear, anxiety, and depression) associated with pathological pain [39, 40].

Over the last few decades, accumulating evidence has shown that pathological pain induces glutamatergic signalling enhancement in the dorsal horn of the spinal cord [41]. This excitatory deregulation is responsible for the spinal wind-up phenomenon and, in turn, for the maintenance of tactile allodynia and hyperalgesia [42]. Conversely, changes in the glutamatergic synapses in forebrain areas linked with allodynia and emotional aspects of chronic pain remain poorly explored. Others and we have demonstrated that enhanced cortical glutamate level may contribute to central sensitization during neuropathic pain [1, 8, 43]. In particular, we showed that 7 days of SNI caused pain behaviour associated with several changes in the prelimbic-infralimbic cortices, including enhanced expression of the transient receptor potential vanilloid type-1 (TRPV1) channel on glutamatergic fibers, with a subsequent increase in extracellular glutamate level [8]. In the present study, we evaluated pain and neuropsychiatric behavioural changes associated with possible bio-molecular, morphological and electrophysiological modifications in the mPFC glutamate synapse at day 30th post-injury. In particular, we quantitated synaptic proteins in the mPFC-derived synaptosome to minimize contamination by non-neural cell derived proteins. SNI mice showed an increase in the expression level of mGluR5 and NMDA-NR2b subunit. These synaptic maladaptive changes in the post-synaptic membrane, similarly to other chronic neuro-psychiatric diseases [44–46], may critically occur following peripheral nerve injury [47]. Accordingly, our results show that expression level of the synaptic scaffolding protein Homer1a (the inducible post-synaptic component responsible for decreasing the aggregation or clustering of the PSD proteins), was increased in SNI animals, while the constitutive Homer1b protein did not change in the same condition. Conversely, we observed that other post-synaptic signal transduction components, such as Shank-1 and PSD-95, which are involved in the pathophysiology of other neurological disorders including schizophrenia [48], were not modified in this model of neuropathic pain. These findings suggest that long-term SNI state may exert a Homer1a-regulated PSD changes that are consistent with the 30 days SNI-induced excitatory synaptic functioning inpaired activity. In fact, even though at 30 days after SNI, animals showed superimposable allodynia and hyperalgesia to the short-term neuropathy (7 days SNI) animals, we observed a decreased - instead of an increased as shown in 7 day SNI - mPFC glutamate level when compared with sham mice. Accordingly, by using single unit extracellular recordings *in vivo*, we found that 30 days-neuropathic mice showed an overall depressed activity of PLC pyramidal neurons, as compared to 7 [8] or 15 days SNI, which instead proved enhanced neuronal activity, as compared to sham animals. Therefore, these findings indicate that long-term injury may be responsible for hypofunctional glutamate signalling at the supraspinal level and, possibly, of the pain-associated negative affective states. Indeed, a fine-tuned change may have occurred in the glutamate synapses since we found here the extracellular levels of D-aspartic acid (D-Asp) significantly enhanced in the mPFC of SNI mice. D-Asp may play a role in synaptic plasticity-mediated processes, such as memory, learning, and pain [49, 50], by acting as an agonist on NMDA receptors through binding to each of the GluN2 subunits [51, 52]. In this study, low level of D-Asp was detectable in physiological conditions, while high level was observed in the mPFC of 30 days-SNI animals. Thus, a possible counterbalance of D-Asp may occur as a consequence of dysregulation of glutamate level following long lasting SNI, which in turn may drive changes in receptor expression as a further adaptive responses. Intriguingly, while PEA reduced pain, as well as the pain-associated affective behavioural changes, it failed to restore the physiological glutamate levels, but normalized D-Asp concentrations in the mPFC.

As we have shown previously [8], our results showed no difference in mPFC GABA levels between sham-operated and SNI animals. Although changes in GABA levels were not appreciable in SNI-induced short or long-term neuropathy, GABA homeostatic concentrations were strongly preserved, possibly to counteract the altered glutamatergic tone.

Similarly to humans, SNI animals remain oversensitive to tactile and thermal stimuli months after injury, with a late development of anxio-depressive syndrome. In this study, animals at 30 days after injury showed mechanical and thermal hypersensitivity, together with depression-like behaviour, cognitive impairments, and obsessive-compulsive-like activities. Daily treatment with the acylethanolamide PEA (or OEA) reduced most of the SNI-induced pain-related behaviours. Even though PEA is effective in neuropathic symptoms in model of relatively short lasting neuropathy (i.e. allodynia is measurable up to 15–20 days after chronic constriction injury of the sciatic nerve, CCI model) [53, 54], we provide here the first evidence that PEA (or OEA) also reduces pain symptoms in a model of debilitating long lasting pain (the SNI model), which is comparable to an advanced stage of neuropathy in humans [55]. It has been shown that different experimental pain models are accompanied by significant changes in the tissue concentrations of analgesic endocannabinoids and PEA [23]. We have previously shown that endogenous PEA levels are down-regulated 7 days after CCI induction in brainstem areas controlling pain, suggesting a role of this acylethanolamide in the development of hyperalgesia in neuropathic pain [23]. In fact, in the current study we observed an increase of PEA levels in the mPFC of 15 days-SNI mice, whilst it was normalized 30 days post nerve injury. All together, these observations indicate that production of PEA might be an adaptive response to neuropathic pain development aimed at counteracting pain transmission or manteinance.

We observed here that PEA also reverted the depression and the impaired performances in discriminative memory associated with long-term neuropathy; importantly, PEA did not affect stereotypical behaviours in the marble burying test. The therapeutic failure of PEA in this latter test, which was applied as an index of anxiety and/or repetitive activity, might suggest that PEA does not engage neural circuits (i.e. striatum) or neurotransmissions (i.e. serotonin or dopamine) shown to be implicated in motor over-activity or obsessive-compulsive disorders [56]. With regard to the resolution of pain and depressive-like syndrome in SNI mice, PEA reversed the Homer1a and Nr2b over-expression in the mPFC of these animals, but not that of mGluR5, which might be spared for the overall maintenance of the excitatory synapses.

Compounds that are able to antagonize the Nr2b subunit selectively are effective in reducing neuropathic pain in animals and patients, by avoiding psychotomimetic effects, as well as motor deficits attributable to the classical analgesics [57]. Moreover, selective Nr2b receptor antagonists produce antidepressant effects (ketamine like) in rodent models [58] and in human subjects [59]. In fact, the crucial role of Nr2b in the the rapid actions of ketamine associated with the downstream regulation of mammalian targets of rapamycin (m-TOR) signalling and BDNF release has been demonstrated [60].

Indeed, in this study, PEA reduced NR2b expression in both sham and neuropathic mice. This finding may indicate a further mechanism of PEA action in order to explain its anti-neuropathic effect as well as its effectiveness in alleviating anxiety/depression-like behaviours. In fact, since the nuclear peroxisome proliferator-activated receptor-α (PPAR-α) has been suggested as its molecular target [61], we can speculate that a 15 days treatment with PEA could be responsible, at least in part, for the Nr2b down-regulation through a genomic mechanism and adds more complexity and value to the so called “entourage effect” of PEA [62].

It is also worthy of note that, the m-TOR signalling, including phospho-Akt and phospho-S6, involved in cells survival, proliferation, and protein synthesis, were up-regulated dramatically in SNI mice and normalized by PEA treatment. Moreover, the SNI-induced reduction of BDNF and the concurrent enhancement of TrkB levels were restored by PEA. In fact, changes in BDNF and subsequent alterations in synaptic plasticity have been shown to contribute to both pain and depression [63, 64]. Intriguingly, the BDNF release by microglia cells in the dorsal horn of spinal cord, has been shown to be associated with the development of tactile allodynia by shifting in neuronal anion gradient in the lamina one nociceptive neurons [65]. Despite the effect of the increased BDNF release in the spinal cord, that also involves the P_2_X_4_-mediated activation of microglia, the few data available at supraspinal level, showed that neuropathic pain is associated with a reduction of the BDNF levels in the cingulate cortex (ACC) [63, 64], according with our data in the mPFC. These changes in supraspinal BDNF level could be related to the depressive-like behaviour rather then directely correlated to the development of tactile allodynia.

These data provide evidence that SNI-associated neuropsychiatric behaviours, particularly the anxious-depressive syndrome, may be related to molecular changes of factors such as BDNF involved in the well-being of neurons. Whether the supraspinal molecular changes are related to peripheral and/or circulating factors (i.e. hormons, cytokines) or driven by a specific brain cell-network (i.e. neurons-astrocytes-microglia) remains unclear. Indeed, this study confirms that supraspinal (at least cortical) microglia are not affected by peripheral nerve injury [38]. However, we observed that PEA induces an increase of hyper-ramified microglia, possibly associated with an alternative activated (non phagocytic) phenotype indicative of an hyper-surveillance state. Moreover, we show that, although it does not alter behaviour in selected tasks, PEA is able to physiologically regulate the substrates production, as well as, neurotransmitters release in brain areas controlling not only pain, but several neurological processes, including cognition, memory and reward. Thus, we can not exclude that PEA may play a physiological role in different neuropsychological functions.

All these results (behaviors and synaptic changes in mPFC) could be simply due to the PEA’s effects in spinal cord or periphery. The possible role of PEA or OEA in decreasing allodynia by also acting within the PL-IL cortex was here suggested by the fact that a single direct microinjection in those forebrain areas exerted a significant anti-allodynic effect. Interestingly, we observed that while OEA microinjection induced a rapid and transient analgesia, probably due to activation/desensitization of capsaicin-sensitive TRPV1 channels [65, 66] PEA exerted a delayed analgesic effects (50–60 min post-injection). This difference suggests that PEA, unlike OEA, might promote via PPARa activation the neo-synthesis of proteins aimed at counteracting pain perception.

Moreover, in order to further clarify the effect of PEA on neurons, we performed a set of *in vitro* experiments. We found that 24 h PEA exposure affected synaptic omeostasis in neuron primary cultures by modifying the expression level of Nr2b and mGluR5 receptors, even though a different modulation of Nr2b, as compared with *in vivo* PEA treatment, was observed. Similar discrepancy already has been observed with antidepressant drugs and might be consequence of differences in dosage, time drug exposure, brain area used or cell cultures [67–69]. Nevertheless, it remains unknown whether the neuropsychiatric changes, including the brain molecular changes, represent effects driven by peripheral injury or prolonged maintenance of the tactile allodynia or both. Thus, based on these findings, we can not roule out that, because of the overall peripheral anti-inflammatory effect of PEA, a “secondary” ameliorating action may be also observed in the mPFC.

## Conclusions

In conclusion, our results demonstrate that long-term peripheral nerve injury causes phenotypic changes in pyramidal neurons of mPFC with a remodeling of the glutamate synapses and modification in the expression of synaptic density molecules. Moreover, we showed that chronic PEA treatment reduced pain and related anxio-depressive behaviour by in part restoring glutamatergic synapse homeostasis. Given the potential role of the mPFC in pain mechanisms, our findings may provide novel insights into neuropathic pain processes and indicate PEA as a new pharmacological tool to treat neuropathic pain and the related negative affective states.

## Additional file

Additional file 1:
**Iba-1 immunoreactivity (Iba-1-ir) is shown in the m-PFC of sham or SNI mice (30 days after injury) following PEA (10 mg/kg, i.p.) or vehicle treatment (A).** Quantitative analysis of total, or activated or hyperbranched, is shown in “B”. Data are expressed as mean ± S.E.M of 3 mice per group. * indicates significant differences compared to sham/vehicle. *P* < 0.05 was considered statistically significant. ANOVA, post hoc Tukey. (PPT 1605 kb)
