# Supplementary figures and images for: Palmitoylethanolamide reduces pain-related behaviors and restores glutamatergic synapses homeostasis in the medial prefrontal cortex of neuropathic mice
Source: Mol Brain. 2015 Aug 12;8:47. doi: 10.1186/s13041-015-0139-5 (PMC4532244; doi:10.1186/s13041-015-0139-5)

## Slide 1
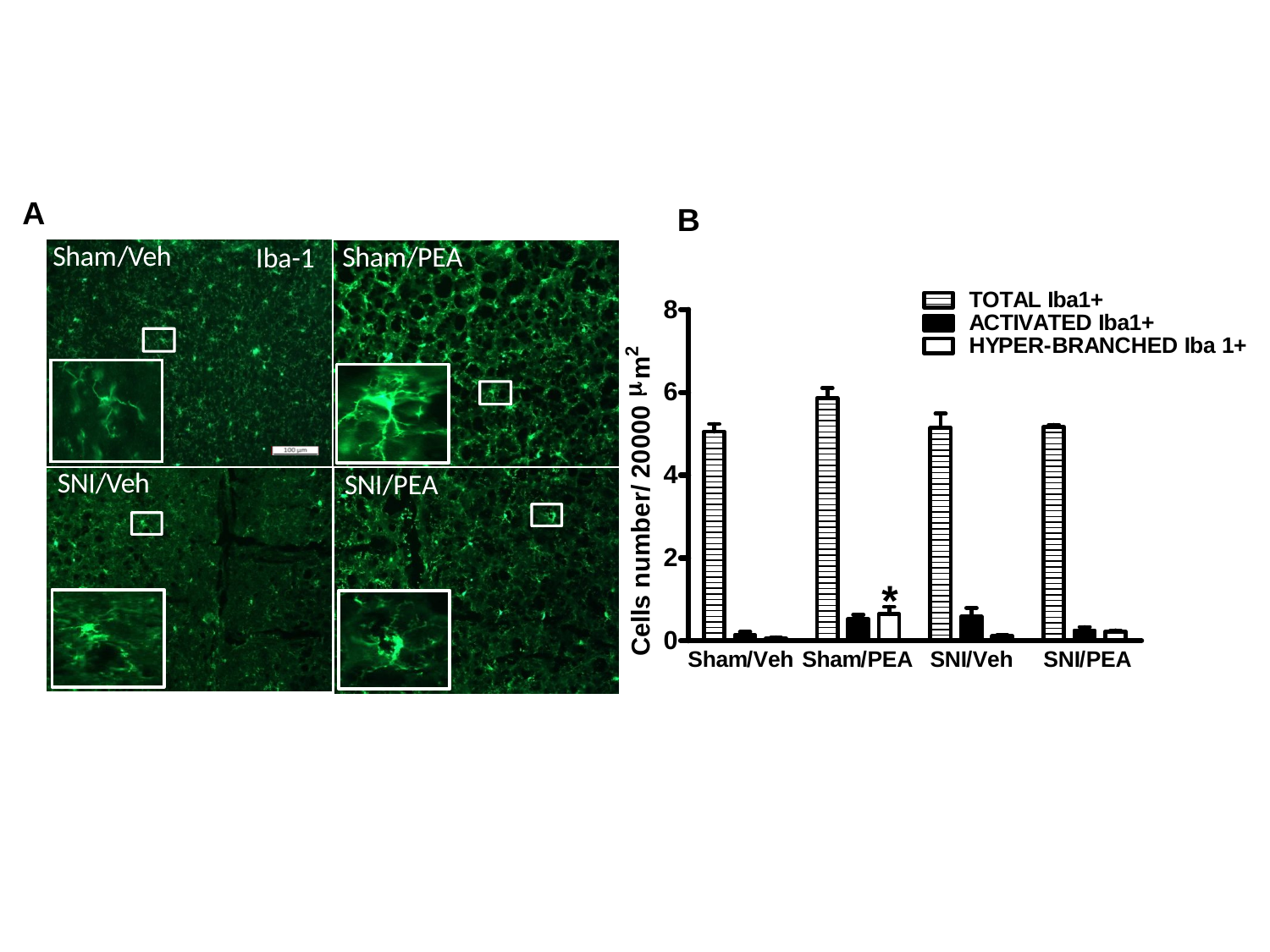

A
B
*

Supplement: Additional file 1: — Iba-1 immunoreactivity (Iba-1-ir) is shown in the m-PFC of sham or SNI mice (30 days after injury) following PEA (10 mg/kg, i.p.) or vehicle treatment (A). Quantitative analysis of total, or activated or hyperbranched, is shown in “B”. Data are expressed as mean ± S.E.M of 3 mice per group. * indicates significant differences compared to sham/vehicle. P < 0.05 was considered statistically significant. ANOVA, post hoc Tukey. (PPT 1605 kb) [file 13041_2015_139_MOESM1_ESM.ppt]
